# Progress in Genito-Urinary Surgery

**Published:** 1904-04-09

**Authors:** 


### Progress in Genito-Urinary Surgery.

(*Continued from Page* 9.)

**Calculous Anuria.**—A. T. Cabot (Boston, Mass.)[4] has discussed the diagnosis and treatment of this condition. This form of anuria is sudden in onset, being due to the stoppage of the ureter by a stone. The suppression of urine may persist from three to ten days before the signs of uræmia become manifest, and sometimes death ensues without uræmic symptoms. Occasionally the stoppage is not persistent, but lasts for several days and then a profuse escape of watery urine occurs. Such cases are explained by the calculus being displaced, *e.g.* into the renal pelvis, or by an alteration of position allowing urine to flow past. Intermittent anuria of this kind is not infrequently associated with hydronephrosis. Calculous anuria may be produced by the simultaneous stoppage of both ureters, in which case, as a rule, the stoppage on one side will be of long standing and the corresponding kidney practically destroyed. In other cases the anuria is the result of the stoppage of the ureter of the only functional active kidney, the other kidney being either absent or destroyed by antecedent disease. There are, however, authentic cases of calculous anuria in which the other kidney has been comparatively healthy. This is, however, very rare, and as a rule the patients have only previously had one active kidney. Legueu analysed 30 cases verified by autopsy and found that the other kidney was absent in three cases, more or less destroyed by calculus in 20 cases, and had the ureter stopped in six. In only one was the other kidney in fairly good condition, and in that there was epithelial proliferation in the tubules. Morris found the second kidney enlarged in two out of 28 cases. A few cases have been reported in which the anuria has ceased with the escape of the stone into the bladder. The reason for this ending being a rare one is that the excretion of urine behind the plug in the ureter ceases and there is no pressure from behind to expel the stone. With the expectant plan of treatment there is a mortality of from 72 to 80 per cent. The results of surgical interference are far better than that. It is of the first importance to determine which ureter has just been stopped by the calculus and can be restored to usefulness by operation. If the pain at the onset of the attack is distinctly localised to one side, especially if that kidney is enlarged and tender, it is fairly clear that operation should be on that side. But often the tension and the pain in the affected kidney are slight and temporary. In the absence of pain and tenderness the course of the ureter should be carefully palpated. A tender area may reveal the position of the calculus. The lower ends of the ureters should be palpated from the rectum or vagina. Radiography, the cystoscope, and catheterisation of the ureters are useful methods of examination. The author relates two cases in which he operated for this condition, but was unable to find the stones. In both the ureters were stroked from above downwards to dislodge the stone if present, though not palpable. Both patients recovered and subsequently calculous material was found to have passed into the bladder. Thus occasionally a cure may be effected by vigorous palpation through the abdominal wall. The lowermost part of the ureter in the male may be reached by a Kraske incision, with displacement of a part of the sacrum. It is to be remembered that it is not necessarily the enlarged kidney which should be approached by operation. Palpation through a laparotomy wound is advised when no evidence can be obtained as to which side is affected. M. L. Kreps [5] has also discussed this subject. He relates the following case: A very stout woman, who had previously suffered a few times from left-sided renal colic, was seized with anuria after another attack of colic. On the third day cystoscopy was performed. The right urethral orifice was missing. With a catheter passed into the left ureter, an impacted stone was felt situated 2 cm. from the vesical orifice. After many attempts, and after the previous injection of hot glycerine, the stone was loosened by means of a thick catheter. Within a quarter of an hour 500 g. of urine were passed, and on the next day 3,000 g. A day later a stone the size of a pea was passed. Cystoscopy again showed the absence of a right ureteral opening. The case was one, therefore, of anuria of a solitary kidney, and not a reflex anuria. The author believes that many cases of so-called reflex anuria are due to the existence of only one kidney, the ureter of which has become blocked. He has, however, observed one genuine case of reflex anuria. The patient had had anuria for one to one and a half days. A stone was loosened in the right ureter as in the last case. A catheter then passed into the left ureter, drew off normal urine. The author formulates the following rules for treatment: (1) In all cases the ureter must be catheterised and the stone moved, if necessary using hot oil or glycerine as an injection into the ureter for the purpose; (2) if a stone is not found in one ureter it must be immediately sought for in the other; (3) if urine does not escape after loosening the stone found in one ureter, the other ureter must be sounded, for the blockage may be bilateral or on one side of ancient date and not the exciting cause of the anuria; (4) if one ever finds on catheterising the ureters, that a ureteral stricture exists, and if there is the slightest suspicion of a renal calculus also being present, then the stricture must be dilated to diminish the danger of the impaction of a stone.

**Intermittent Hydronephrosis.**—M. H. Richardson [6] reports a case of this affection in a man of 23. For six months he had had pain in the left side and there was a large tender mass in the position of the left kidney. Within five days the mass disappeared. The tumour re-formed. An incision was made over it. The hydronephrosis was caused by a sharp bend in the ureter at its origin at the pelvis of the kidney. An incision, about an inch in length, was made, extending through the pelvis of the kidney and along the ureter over the angular bend at its junction with the pelvis. This longitudinal wound was converted into a transverse one by suturing. The patient was well and able to work at the end of a month.

**Cystoscopy.**—Thomas Wilson [7] discusses the technique and uses of cystoscopy by Kelly's method. In gynæcological practice many of the patients have symptoms of derangement of the urinary organs. In most cases these symptoms are due to disturbance of function caused by affections of the uterus or its appendages, and subside when the latter affections are efficiently treated. In other cases the symptoms are the result of disease primarily or chiefly affecting the urinary organs; or, again, disease of the urinary organs may co-exist with disease of the generative organs. In such cases the remedies for the latter affections may entirely fail to relieve those of the former. For the elucidation of all these cases visual inspection of the interior of the bladder affords most valuable help. The inspection may be made with an electric cystoscope or by the Kelly method. The latter was placed on a firm basis and fully described by Kelly in January 1894. The author has used the method on 250 occasions. The specula used, consisting of metallic tubes of various sizes provided with a handle, are well known. A forehead light or reflector is used for the illumination. The patient lies on the back with the knees well drawn up and separated, the bladder having been emptied with a catheter. The size of the urethra is measured with a conical calibrater, and the appropriate speculum selected and passed. The sacrum is then raised eight or twelve inches above the table with sand-bags, and the legs flexed on the abdomen and supported by assistants. The bladder then fills with air. In withdrawing the speculum the urethra is examined. At the conclusion of the examination the air must be removed, after lowering the sacrum, by passing a catheter. If this is not done, severe colicky pains will be experienced in the lower abdomen. In at least 90 per cent. of his cases the author found it unnecessary to use either general or local anæsthesia. The knee-elbow position is more efficacious for distending the bladder, but is objectionable on æsthetic grounds and because it quickly tires the patient.

The author uses it only when the elevation of the sacrum fails to produce ballooning of the bladder. As compared with electrical cystoscopy with the Nitze instrument the Kelly method is considered easier to learn, and has the further advantages that it can be carried out when the sphincter acts imperfectly or when bleeding is going on. The apparatus is simple, easily sterilised, and comparatively inexpensive.

[4] Ann. of Surg., Oct. 1903, p. 503. [5] (Abst.) Zentralb. f. Chir., No. 46, Nov. 1903, p. 1283. [6] Boston Med. and Surg. Jour., Jan. 14, 1904. [7] Lancet, Jan. 9, 1904, p. 79.

*(To be continued.)*

